# Association between ethnicity and migration status with the prevalence of single and multiple long-term conditions in UK healthcare workers

**DOI:** 10.1186/s12916-023-03109-w

**Published:** 2023-11-30

**Authors:** Winifred Ekezie, Christopher A. Martin, Rebecca F. Baggaley, Lucy Teece, Joshua Nazareth, Daniel Pan, Shirley Sze, Luke Bryant, Katherine Woolf, Laura J. Gray, Kamlesh Khunti, Manish Pareek, on behalf of the UK-REACH study collaborative group, Laura Nellums, Anna L. Guyatt, Catherine John, I Chris McManus, Ibrahim Abubakar, Amit Gupta, Keith R. Abrams, Martin D. Tobin, Louise Wain, Sue Carr, Edward Dove, David Ford, Robert Free

**Affiliations:** 1 Diabetes Research Centre, University of Leicester, Leicester, UK; 2 Centre for Ethnic Health Research, University of Leicester, Leicester, UK; 3 National Institute for Health and Care Research (NIHR), Applied Research Collaboration East Midlands (ARC EM), Leicester, UK; 4 School of Social Sciences and Humanities, Aston University, Birmingham, UK; 5 Department of Infection and HIV Medicine, University Hospitals of Leicester, NHS Trust, Leicester, UK; 6 Department of Respiratory Sciences, University of Leicester, Leicester, UK; 7 NIHR Leicester Biomedical Research Centre (BRC), University of Leicester, Leicester, UK; 8 Development Centre for Population Health, University of Leicester, Leicester, UK; 9 Department of Population Health Sciences, University of Leicester, Leicester, UK; 10 Li Ka Shing Centre for Health Information and Discovery, Oxford Big Data Institute, University of Oxford, Oxford, UK; 11 Cardiovascular Research Centre, University of Leicester, Glenfield Hospital, Leicester, UK; 12 UCL Medical School, Gower Street, London, UK

**Keywords:** Ethnic minorities, Migrants, Morbidity, Multiple chronic conditions, Multimorbidity, Comorbidity, Healthcare workers, United Kingdom

## Abstract

**Background:**

Healthcare workers’ (HCW) well-being has a direct effect on patient care. However, little is known about the prevalence and patterns of long-term medical conditions in HCWs, especially those from ethnic minorities. This study evaluated the burden of multiple long-term conditions (MLTCs), i.e. the presence of two or more single long-term conditions (LTCs), among HCWs in the United Kingdom (UK) and variation by ethnicity and migration status.

**Methods:**

We used baseline data from the UK-REACH cohort study collected December 2020–March 2021. We used multivariable logistic regression, adjusting for demographic, occupational and lifestyle factors to examine the relationship between self-reported LTCs/MLTCs and ethnicity, migration status and time since migration to the UK.

**Results:**

Of 12,100 included HCWs, with a median age of 45 years (IQR: 34–54), 27% were overseas-born, and 30% were from non-White ethnic groups (19% Asian, 4% Black, 4% Mixed, 2% Other). The most common self-reported LTCs were anxiety (14.9%), asthma (12.2%), depression (10.7%), hypertension (8.7%) and diabetes (4.0%). Mental health conditions were more prevalent among UK-born than overseas-born HCWs for all ethnic groups (adjusted odds ratio (aOR) using White UK-born as the reference group each time: White overseas-born 0.77, 95%CI 0.66–0.95 for anxiety). Diabetes and hypertension were more common among Asian (e.g. Asian overseas, diabetes aOR 2.97, 95%CI 2.30–3.83) and Black (e.g. Black UK-born, hypertension aOR 1.77, 95%CI 1.05–2.99) groups than White UK-born. After adjustment for age, sex and deprivation, the odds of reporting MLTCs were lower in most ethnic minority groups and lowest for those born overseas, compared to White UK-born (e.g. White overseas-born, aOR 0.68, 95%CI 0.55–0.83; Asian overseas-born aOR 0.75, 95%CI 0.62–0.90; Black overseas-born aOR 0.52, 95%CI 0.36–0.74). The odds of MLTCs in overseas-born HCWs were equivalent to the UK-born population in those who had settled in the UK for ≥ 20 years (aOR 1.14, 95%CI 0.94–1.37).

**Conclusions:**

Among UK HCWs, the prevalence of common LTCs and odds of reporting MLTCs varied by ethnicity and migrant status. The lower odds of MLTCs in migrant HCWs reverted to the odds of MLTCs in UK-born HCWs over time. Further research on this population should include longitudinal studies with linkage to healthcare records. Interventions should be co-developed with HCWs from different ethnic and migrant groups focussed upon patterns of conditions prevalent in specific HCW subgroups to reduce the overall burden of LTCs/MLTCs.

**Supplementary Information:**

The online version contains supplementary material available at 10.1186/s12916-023-03109-w.

## Background

Nearly two million people in the United Kingdom (UK) are employed in the healthcare workforce [1]; 16.3% of NHS staff in England are non-British [2], and around 21.0% are from non-White ethnic groups [3]. Few studies have examined the burden of single long-term conditions (LTCs), often in relation to COVID-19, but none have reported on the occurrence of multiple long-term conditions (MLTCs) in healthcare workers (HCWs). Evidence on this topic is vital as the health of HCWs is critical not only for their own well-being but also for their ability to render good quality services to their patients and future service planning. A high burden of long-term health conditions may result in increased sickness absence, leading to understaffing, delays in patient care and an additional workload for other staff. At an organisational level, sickness absence in HCWs is associated with a financial cost through paid sick leave and covering rota gaps with locum workers. Hence, poor HCW health status can affect patient care, safety and satisfaction through decreased healthcare access and increased healthcare costs [4]. In the UK, this problem is compounded by the current NHS staffing crisis with estimated shortages of 12,000 hospital doctors and over 50,000 nurses and midwives [5].

When examining the health profiles of HCWs, it is crucial to consider the unique occupational hazards this group faces. This was brought into focus during the COVID-19 pandemic, with evidence suggesting a higher risk of infection and associated adverse outcomes from COVID-19 compared to the general population [6, 7]. Notably, the risk of infection and adverse outcomes were observed to be greater for HCWs from ethnic minority groups [2, 8], who may also be at increased risk of long-term COVID-19 sequelae and poor mental health outcomes such as anxiety, depression, and post-traumatic stress [9, 10].

Multiple long-term conditions (MLTCs) describe the co-existence of two or more chronic conditions (e.g. cardiovascular diseases, cancer, diabetes, and mental health conditions) [10]. MLTCs are a major public concern, with an estimated 14 million people in England living with MLTCs, including around 20% of those aged between 25 and 64 [11]. Previous studies in the general population have shown a higher prevalence of MLTCs in ethnic minority groups [12–14]. Additionally, non-refugee migrants have a lower prevalence of MLTCs than non-migrants in the early years following migration; however, the risk of developing MLTCs increases as years settled in a new country increase [12–15]. An overview of predominant chronic conditions, patterns of their co-occurrence, and health outcomes among migrants in different work sectors (including HCWs) has not been extensively explored [16] and to our knowledge, no study has examined whether there are differences in the prevalence of LTCs/MLTCs within ethnic groups according to migration status among HCWs. We, therefore, aimed to evaluate the presence of LTCs and MLTCs among UK HCWs and whether these vary by ethnicity and migration status. Our null hypothesis was that the prevalence of LTCs/MLTCs would not vary according to ethnicity and migration status in a cohort of UK HCWs.

## Methods

### Overview

The *U*nited *K*ingdom *R*esearch study into *E*thnicity *A*nd **C**OVID-19 diagnosis and outcomes in *H*ealthcare workers (UK-REACH) is a programme of work established to investigate the disproportionate impact of the COVID-19 pandemic on HCWs from ethnic minority groups. For the present analysis, we used data collected in the baseline questionnaire of the UK-REACH prospective nationwide cohort study. The questionnaire was electronically administered between December 2020 and March 2021. For a full description of the study methods and the cohort, see the study cohort paper [17]. No personally identifiable information was collected in the questionnaire. Details of the measures included in the questionnaire can be found in the data dictionary (https://www.uk-reach.org/main/data-dictionary/).

The study was approved by the Health Research Authority (Brighton and Sussex Research Ethics Committee; ethics reference: 20/HRA/4718) and registered with ISRCTN (Reference ISRCTN 11811602).

### Study population and recruitment

We included HCWs (including ancillary workers in a healthcare setting) aged 16 years or older and/or registered with one of seven professional healthcare regulators (with registrants from across the four countries of the UK, see Additional file 1: Text S2). Recruitment details have been described elsewhere [17, 18]. Briefly, Professional regulators sent out emails and newsletters with a link to the study website to their registrants. Recipients then had to read the email, navigate to the study website and register to create a profile. Following the creation of a study profile, potential participants were asked to read a participant information sheet and then, if they were willing, consent to participate in the study*.*The sample was supplemented with direct recruitment from participating NHS Trusts. Here, we report participant response rates at each stage of this multi-step process as recommended by the Checklist for Reporting Results of Internet E-Surveys (CHERRIES) [19].

### Exposure

Our primary exposures of interest were self-reported ethnicity and migration status. We asked participants to select their ethnicity from the 18 standardised groups used by the Office for National Statistics (ONS) in the 2021 Census for England and Wales. For analysis, the categories were collapsed into five aggregated ethnic groups (White, Asian, Black, Mixed, and Other) also used by the ONS. We used migrant status (whether the participant was born in the UK or overseas) to split each ethnic group in two, resulting in a ten-level categorical variable (see Additional file 2: Table S1). White UK-born was used as the reference group in regression models.

### Outcome measures

We examined the relationship between ethnicity and migration status with the following outcomes: (i) presence of LTCs (binary variable where 1 = reports having the condition and 0 = does not report having the condition); (ii) MLTCs (binary variable where 1 = reports the co-occurrence of two or more LTCs and 0 = does not report the co-occurrence of two or more LTCs); and (iii) number of LTCs (continuous variable − a count of the number of LTCs reported). We derived these outcome measures from a single questionnaire item. Study participants were asked to indicate whether they had any of the following health conditions (drawn from a list included in the Wellcome Trust COVID-19 questionnaire): [20] organ transplants; diabetes (type I or II); heart disease or heart problems; hypertension; overweight; stroke; kidney disease; liver disease; anaemia; asthma; other lung condition such as chronic obstructive pulmonary disease (COPD), bronchitis or emphysema; cancer; conditions affecting the brain and nerves (e.g. dementia, Parkinson’s, multiple sclerosis); a weakened immune system or reduced ability to deal with infections (as a result of a disease or treatment); depression; anxiety; psychiatric disorder.

We made a priori decisions based on the literature and expert opinion about which LTCs to include in the analysis [21]. We elected not to examine: overweight (instead, we used self-reported body mass index (BMI) as a covariate); anaemia (we lacked information on the severity and aetiology of the condition and felt this would introduce significant heterogeneity among those selecting this option); and psychiatric disorder (the wording of this option was confusing given information on anxiety and depression — both psychiatric disorders — was collected separately). We included all other conditions in deriving the long-term condition count and the binary MLTCs outcome variable (≥2 vs <2 LTCs).

### Covariates

We collected information on the following potential demographic/occupational confounders of the relationship between ethnicity and migration status with LTCs in our cohort: age, sex, occupation, and Index of Multiple Deprivation (IMD) of the residential area [22]. We also collected data relating to health and lifestyle factors, which might mediate differences in outcome measures according to ethnicity or migration status. These included: smoking status, consumption of alcohol (units per week), physical activity index (PAI) and BMI (using ethnicity-specific thresholds) [23]. Finally, we collected information on the time since migration for those who indicated they were not born in the UK. A description of each variable and its derivation can be found in Table S1.

### Statistical analysis

We excluded those who did not provide information on the exposures (i.e. ethnicity or migration status) and outcomes (i.e. long-term condition status) from the analysis. Categorical variables were summarised as frequencies and percentages, and non-normally distributed continuous variables as the median and interquartile range (IQR). We report the demographic factors (e.g. sex, age, IMD), the frequency, prevalence, and 95% confidence interval (95%CI) of each included long-term condition and the frequency and percentage of the five most prevalent LTCs by ethnicity and migration status. We examined the patterns of co-occurrence of LTCs in UK-born and migrant groups using heatmaps.

Multiple imputation was used to replace missing data in all logistic and negative binomial regression models. Rubin’s Rules were applied to combine parameter estimates and standard errors from 10 imputed datasets into a single set of results. Imputation models included all covariates used in the analysis as well as the exposures and the outcome of interest.

We used logistic regression to determine the association of ethnicity and migration status with (i) the five most prevalent LTCs adjusting for demographic and occupational factors), and (ii) the presence of MLTCs first adjusting for demographic and occupational factors, and second with additional adjustment for health and lifestyle factors. We undertook the sequential adjustment in (ii) to examine how differences in health and lifestyle factors by ethnicity/migration status might contribute to ethnic differences in MLTCs (by examining how odds ratios change between models). We also assessed the relationship between time since migration to the UK and the presence of MLTCs using logistic regression adjusting for demographic factors and occupation. Results from these models are presented as adjusted odds ratios (aORs) and 95%CIs.

### Sensitivity analyses

We examined the association of ethnicity and migration status with the count of co-occurring LTCs after adjustment for demographic and occupational factors and health and lifestyle factors using negative binomial regression models (as the outcome data were overdispersed). Results are provided as adjusted rate ratios (aRRs) and 95%CIs.

## Results

### Recruitment and formation of the study sample

The detailed formation of the study cohort is shown in Additional file 3: Fig. S1. A total of 12,100 HCWs were included in this primary analysis.

### Description of the analysed study sample

Table 1 shows the demographic and occupational characteristics of the analysed sample. The median age was 45 years (IQR: 34–54); most respondents were female (76%). 29.8% of respondents were overseas-born, and 30% were from non-White ethnic groups (19.2% Asian, 4.3% Black, 4.2% Mixed, 2.1% Other). The socioeconomic status of the study sample was higher than the UK population average, with almost half the sample (46.7%) residing in the least deprived areas (IMD quintiles 4 and 5) and only 8.8% in the most deprived quintile. Allied Health Professionals (AHPs, including healthcare scientists, ambulance workers, pharmacy staff and those in optical roles) were the largest group of participating HCWs by occupation (42%), followed by those in medical (23%) or nursing (21%) roles.

**Table 1:** Description of the UK-REACH cohort — results from the baseline questionnaire

**Variable**	**Description** **Total *n* = 12,100**
**Age in years**, median (IQR)	45 (34–54)
Missing	62 (0.5)
**Sex**	
Male	2876 (23.8)
Female	9199 (76.0)
Missing	25 (0.2)
**Ethnicity and migration status**	
White UK-born	7444 (61.5)
White overseas-born	1048 (8.7)
Asian UK-born	834 (6.9)
Asian overseas-born	1492 (12.3)
Black UK-born	152 (1.3)
Black overseas-born	369 (3.1)
Mixed UK-born	383 (3.2)
Mixed overseas-born	130 (1.1)
Other UK-born	45 (0.4)
Other overseas-born	203 (1.7)
**Occupation**	
Medical	2745 (22.7)
Nursing	2489 (20.6)
Allied Health Professional^a^	5057 (41.8)
Dental	734 (6.1)
Admin/estates/other	642 (5.3)
Missing	433 (3.6)
**Index of multiple deprivation quintile**	
1 (most deprived)	1063 (8.8)
2	1758 (14.5)
3	2204 (18.2)
4	2593 (21.4)
5 (least deprived)	3064 (25.3)
Missing	1418 (11.7)
**Body mass index (kg/m**^**2**^**)**^b^	
Underweight	151 (1.3)
Healthy weight	4512 (37.3)
Overweight	3645 (30.1)
Obesity class 1	1678 (13.9)
Obesity class 2	604 (5.0)
Obesity class 3	323 (2.7)
Missing	1187 (9.8)
**Physical activity index**	
Active	3993 (33.0)
Moderately active	2568 (21.2)
Moderately inactive	2567 (21.2)
Inactive	2407 (19.9)
Missing	565 (4.7)
**Smoking status**	
Never smoker	8736 (72.2)
Ex-smoker	2659 (22.0)
Current smoker	599 (5.0)
Missing	106 (0.9)
**Units of alcohol per week**	
None	5007 (41.4)
1–7	3838 (31.7)
8–14	1835 (15.2)
15–21	824 (6.8)
>28	536 (4.4)
Missing	60 (0.5)

^a^Includes those working in pharmacy, optical, healthcare scientist and ambulance roles

^b^Using ethnicity-specific cut-offs (see the “ Methods” section for details)

All data are *n* (%) unless otherwise stated

*IQR* Interquartile range

### Prevalence of long-term conditions (LTCs)

The prevalence of each long-term condition is listed in Table 2. Anxiety (*n*=1804, 14.9%, 95%CI 14.3–15.6%), asthma (*n*=1471, 12.2%, 95%CI 11.6–12.8%), depression (*n*=1296, 10.7%, 95%CI 10.2–11.3%), hypertension (*n* = 1056, 8.7%, 95%CI 8.2–9.2%) and diabetes (*n*=486, 4.0%, 95%CI 3.7–4.4%) were the most prevalent conditions.

**Table 2:** Prevalence of long-term conditions (LTCs) reported by UK healthcare workers in the baseline questionnaire of the UK-REACH cohort study

**Long-term condition**	**Frequency** **Total *n* = 12,100**	**% prevalence (95%CI)**
Anxiety	1804	14.9 (14.3–15.6)
Asthma	1471	12.2 (11.6–12.8)
Depression	1296	10.7 (10.2–11.3)
Hypertension	1056	8.7 (8.2–9.2)
Diabetes	486	4.0 (3.7–4.4)
Immunosuppression	413	3.4 (3.1–3.8)
Heart disease	321	2.7 (2.4–3.0)
Cancer	111	0.9 (0.8–1.1)
Neurological	110	0.9 (0.8–1.1)
Other lung disease	109	0.9 (0.8–1.1)
Kidney disease	91	0.8 (0.6–0.9)
Liver disease	63	0.5 (0.4–0.7)
Stroke	44	0.4 (0.3–0.5)
Organ transplant	15	0.1 (0.1–0.2)

*95%CI* 95% confidence interval

#### Association of prevalent long-term conditions (LTCs) with ethnicity and migration status

**Fig. 1:**
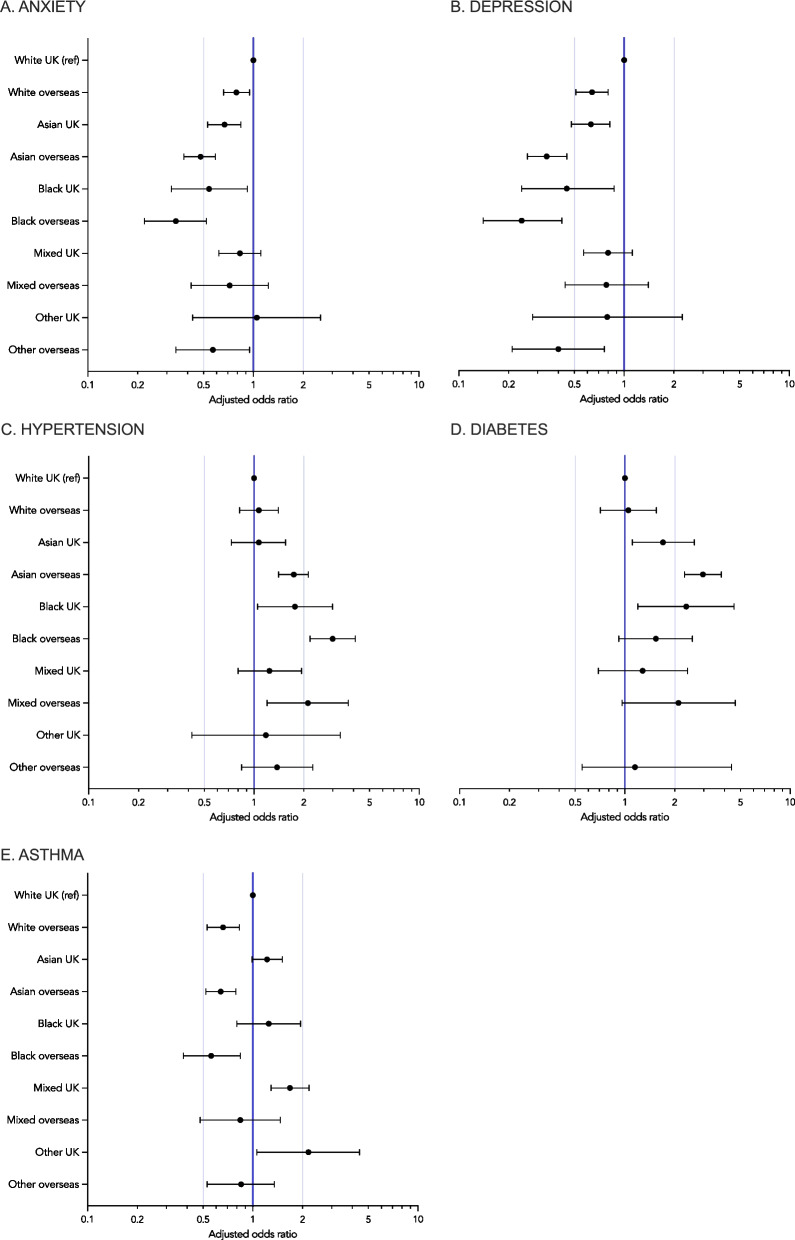
The association of ethnicity and migration status with the five most prevalent long-term health conditions reported by healthcare workers in the UK-REACH study. Associations are derived from logistic regression models presented as odds ratios (circles) and 95% confidence intervals (spiked bars). White UK-born is the reference group. Odds ratios are adjusted for age, sex, index of multiple deprivation quintile and occupation. *N*=12,100 for all models. Panel **D**: results for the association between the “Other UK-born” group and diabetes are omitted due to low numbers (<5) in this group producing a very wide 95% confidence interval range (0.07–4.13)

Additional file 4: Table S2, shows the frequency (%) of participants reporting the five most common conditions, stratified by ethnic/migrant group. Figure 1 shows odds ratios for the association of ethnicity and migration status with these conditions after adjustment for sociodemographic/occupational factors (Fig. S 2 shows the same figure, including the aOR and 95%CI values as text).

The pattern of association between ethnicity and migrant status with reporting a mental health condition was similar for anxiety and depression (Fig. 1A and B, Additional file 5: Fig. S2A and S2B). Compared to White UK-born HCWs, the odds of reporting either mental health condition were lower in most other studied ethnic/migrant groups. Within ethnic groups, the odds ratios for mental health conditions for overseas-born HCWs tended to be lower than their UK-born counterparts when compared to the White UK-born group (e.g. for anxiety: Asian UK-born aOR 0.67, 95%CI 0.53–0.84; Asian overseas-born aOR 0.48, 95%CI 0.38–0.59).

The relationship between ethnicity and migrant status with reported diabetes and hypertension displayed a different pattern, with Asian and Black HCWs having higher odds of these conditions than White HCWs (Fig. 1C and D, Additional file 5: Fig. S2C and S2D). Within each ethnic group, odds ratios of developing diabetes and hypertension for overseas-born HCWs tended to be higher than their UK-born counterparts when compared to the White UK-born group with the exception of Black ethnic groups and diabetes (e.g. for hypertension Black UK-born aOR 1.77, 95%CI 1.05–2.99; Black overseas-born aOR 2.99, 95%CI 2.18–4.10; for diabetes: Asian UK-born aOR 1.70, 95%CI 1.11–2.63; Asian overseas-born aOR 2.97, 95%CI 2.30–3.83).

Figure 1E and Additional file 5: Fig. S2E show the differences between ethnic and migrant groups for odds of reporting asthma. Adjusted odds ratios were greater than 1 for all UK-born ethnic minority groups in comparison to the White UK group but reached statistical significance only in the Mixed and Other ethnic groups. In contrast, adjusted odds ratios for all overseas groups were less than one, and this was statistically significant for the White, Asian and Black groups.

### Co-occurrence of long-term conditions (LTCs)

Heatmaps illustrating the pattern of prevalence of two LTCs according to migration status (UK-born, overseas-born) are shown in Additional file 6: Fig. S3. For UK-born respondents, anxiety and depression had higher co-occurrence (48.3%) than in overseas-born respondents (37.5%). There was a high co-occurrence of hypertension with other LTCs in both UK-born and overseas-born, including organ transplant, diabetes, heart disease, stroke, kidney disease, liver disease and lung diseases other than asthma (20.2–47.1% for UK-born respondents, 10.0–50.0% for overseas-born respondents). The co-occurrence of diabetes with liver disease (17.8% for UK-born, 66.7% for overseas-born) and hypertension with kidney disease (34.4% for UK-born, 50.0% for overseas-born) was more marked for overseas-born HCWs than for UK-born HCWs, but care must be taken not to overinterpret the observed patterns of co-occurring LTCs due to small numbers reporting certain conditions (e.g. organ transplant *n*=15, stroke *n*=44).

#### Association of multiple long-term conditions (MLTCs) with ethnicity and migration status

Table 3 shows results from two logistic regression analyses to explore factors associated with reporting two or more LTCs listed in Table 1. The first regression (A) includes demographic and occupational covariates only, while the second (B) additionally includes health and lifestyle covariates. Overall, the prevalence of reported MLTCs was 15.0%. Raw prevalence was lower in all ethnic/migrant groups compared to the White UK-born reference group. When adjusting for sociodemographic covariates only (regression A), significantly reduced odds of MLTCs were noted in the White, Asian, Black and Other overseas-born groups compared to the White UK-born group (White overseas-born aOR 0.68, 95%CI 0.55–0.83; Asian overseas-born aOR 0.75, 95%CI 0.62–0.90; Black overseas-born aOR 0.52, 95%CI 0.36–0.74). This effect was more pronounced (in all groups apart from White overseas-born) after adjustment for health and lifestyle covariates (regression B), where significant differences were also found for Asian UK-born and Black UK-born groups (White overseas-born aOR 0.69, 95%CI 0.56–0.85; Asian overseas-born aOR 0.63, 95%CI 0.52–0.77; Black overseas-born aOR 0.39, 95%CI 0.27–0.56; Asian UK-born aOR 0.69, 95%CI 0.54–0.87; Black UK-born aOR 0.47, 95%CI 0.28–0.79).

**Table 3:** The association of ethnicity, migration status, sociodemographic and health and lifestyle factors with MLTCs (≥2 long-term conditions): (A) adjusted for ethnicity, migration status and sociodemographic covariates only, and (B) additionally adjusted for health and lifestyle covariates (*n*=12,100)

		**A**		**B**	
**Variable**	***N* reporting MLTCs/*N* total (%)** **1817/12,100 (15.0)**	**Adjusted odds ratio (95%CI)**	***P* value**	**Adjusted odds ratio (95%CI)**	***P* value**
**Ethnicity/migration status**					
White UK-born	1267/7444 (17.0)	Ref	-	Ref	-
White overseas-born	122/1048 (11.6)	0.68 (0.55–0.83)	<0.001	0.69 (0.56–0.85)	<0.001
Asian UK-born	95/834 (11.4)	0.80 (0.63–1.00)	0.05	0.69 (0.54–0.87)	0.002
Asian overseas-born	171/1492 (11.5)	0.75 (0.62–0.90)	0.002	0.63 (0.52–0.77)	<0.001
Black UK-born	18/152 (11.8)	0.65 (0.39–1.07)	0.09	0.47 (0.28–0.79)	0.004
Black overseas-born	35/269 (9.5)	0.52 (0.36–0.74)	<0.001	0.39 (0.27–0.56)	<0.001
Mixed UK-born	63/383 (16.5)	1.13 (0.85–1.50)	0.39	1.10 (0.82–1.47)	0.54
Mixed overseas-born	19/130 (14.6)	0.94 (0.57–1.55)	0.81	0.85 (0.51–1.42)	0.52
Other UK-born	7/45 (15.6)	1.05 (0.47–2.39)	0.90	0.83 (0.36–1.93)	0.67
Other overseas-born	20/203 (9.9)	0.61 (0.38–0.98)	0.04	0.47 (0.29–0.77)	0.002
**Age**, per decade increase	-	1.11 (1.06–1.16)	<0.001	1.08 (1.03–1.13)	0.002
**Sex**					
Male	423/2876 (14.7)	Ref	-	Ref	-
Female	1394/9199 (15.2)	0.86 (0.76–0.98)	0.02	0.89 (0.78–1.02)	0.08
**Occupation**					
Medical	280/2745 (10.2)	Ref	-	Ref	-
Nursing	537/2489 (21.6)	2.13 (1.79–2.53)	<0.001	1.55 (1.29–1.86)	<0.001
Allied Health Professional^a^	689/5,057 (13.6)	1.28 (1.09–1.51)	0.003	1.15 (0.97–1.36)	0.11
Dental	99/734 (13.5)	1.32 (1.02–1.70)	0.04	1.10 (0.85–1.43)	0.47
Admin/estates/other	130/642 (20.3)	1.98 (1.56–2.52)	<0.001	1.40 (1.09–1.79)	0.009
**IMD quintile**					
1 (most deprived)	199/1063 (18.7)	1.21 (1.00–1.47)	0.05	1.11 (0.92–1.35)	0.28
2	281/1758 (16.0)	1.01 (0.84–1.20)	0.96	0.99 (0.83–1.19)	0.94
3	367/2204 (16.7)	Ref	-	Ref	-
4	373/2593 (14.4)	0.85 (0.73–0.99)	0.04	0.89 (0.76–1.04)	0.16
5 (least deprived)	387/3064 (12.6)	0.73 (0.63–0.86)	<0.001	0.80 (0.68–0.94)	0.005
**Body mass index (kg/m**^**2**^**)**^b^					
Underweight	13/151 (8.6)			0.92 (0.51–1.64)	0.77
Healthy weight	419/4512 (9.3)			Ref	-
Overweight	532/3645 (14.6)			1.59 (1.39–1.82)	<0.001
Obesity class 1	352/ 1678 (21.0)			2.29 (1.96–2.69)	<0.001
Obesity class 2	175/604 (29.0)			3.17 (2.56–3.92)	<0.001
Obesity class 3	117/323 (36.2)			4.00 (3.10–5.17)	<0.001
**Physical activity index**					
Active	435/3993 (10.9)			Ref	-
Moderately active	372/2568 (14.5)			1.24 (1.06–1.44)	0.006
Moderately inactive	438/2567 (17.1)			1.34 (1.15–1.56)	<0.001
Inactive	495/2407 (20.6)			1.60 (1.38–1.86)	<0.001
**Smoking status**					
Never smoker	1140/8736 (13.1)			Ref	-
Ex-smoker	510/2659 (19.2)			1.28 (1.13–1.45)	<0.001
Current smoker	15/599 (25.9)			1.73 (1.41–2.13)	<0.001
**Units of alcohol per week**					
None	850/5,007 (17.0)			Ref	-
1–7	482/3838 (12.6)			0.72 (0.63–0.82)	<0.001
8–14	241/1835 (13.3)			0.71 (0.60–0.83)	<0.001
15–21	121/824 (14.7)			0.70 (0.56–0.88)	0.002
>28	106/536 (19.8)			0.89 (0.70–1.13)	0.34

^a^Includes those working in pharmacy, optical, healthcare scientist and ambulance roles

^b^Using ethnicity-specific cut-offs (see the “ Methods” section and Supplementary Table S1 for details). The percentages in the lefthand column are calculated based on those with complete data in the relevant field

*95%CI* 95% confidence interval, *IMD* Index of multiple deprivation, *Ref* Reference group

Additional file 7: Table S3, shows the results of a negative binomial regression to explore the association of ethnicity, migration status, sociodemographic and health factors with long-term condition count. Results suggested the same associations as found in the logistic regressions (Table 3), albeit with a stronger relationship suggested for the effect of sex, with women reporting MLTCs at a lower rate than men.

### Multiple long-term conditions (MLTCs) and duration of UK settlement

**Fig. 2:**
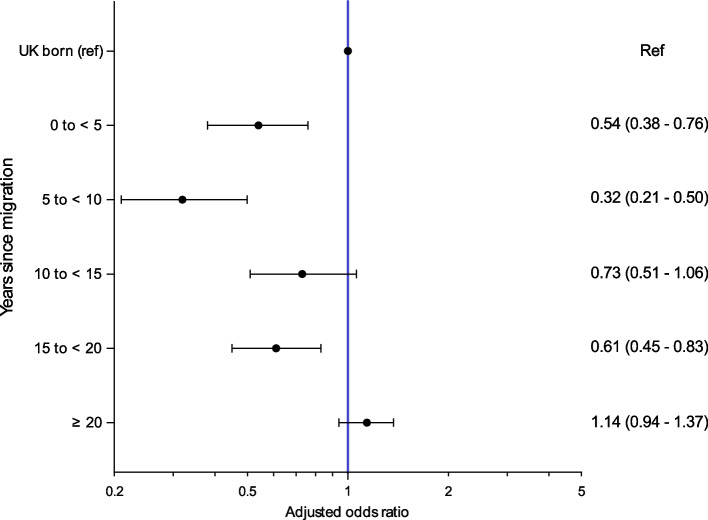
Relationship between time since migration to the UK and risk of multiple long-term conditions (≥2 long-term conditions) expressed as odds ratios stratified by duration resident in the UK, compared to UK-born study participants (*n*=12,100). Odds ratios are adjusted for ethnicity, sociodemographic, health and lifestyle covariates. ref, reference group. Eighty-five missing values for years in the UK were imputed

Figure 2 shows how reporting of MLTCs changes for migrants by the duration of settlement in the UK. Odds ratios are adjusted for demographic factors and occupation. Migrants had significantly lower odds of reporting MLTCs upon arrival in the UK up to ≥20 years of residence, after which odds of MLTCs do not significantly differ from the UK-born respondents.

## Discussion

This study is the first to present national data on the self-reported prevalence of LTCs and MLTCs among HCWs in the UK. The five most prevalent conditions in our cohort were anxiety (14.9%), asthma (12.2%), depression (10.7%), hypertension (8.7%) and diabetes (4.0%). The prevalence of MLTCs was 15.0%. Our study is also the first to demonstrate differences in the odds of reporting these conditions and of reporting MLTCs by a combination of ethnicity and migration status and show that the odds of reporting MLTCs in overseas-born HCWs increase to match the odds of UK-born HCWs after 20 years in the UK.

Our study found ethnicity and migration status to be associated with the self-reporting of common LTCs. Mental health conditions were less prevalent in ethnic minority groups, particularly those born overseas, compared to the White UK-born group. Another UK HCW cohort study found that Black HCWs were slightly less likely to screen positive for depression (using Patient Health Questionnaire-9) than White HCWs; however, they found no differences for other ethnic groups and no ethnic differences in the proportion screening positive for anxiety and post-traumatic stress disorder (PTSD) [24]. It should be noted that there is some evidence that in the general population, those from ethnic minority groups are more likely to have undiagnosed and untreated mental illness [25, 26], a factor which may have affected our results. With regards to the overall prevalence of mental health conditions, it should be remembered that the cohort in this study was recruited during the COVID-19 pandemic; hence the predominance of mental health conditions might be more related to higher levels of workplace stress, burnout and other “work‑related” mental illness, which was significantly higher during the pandemic [27].

Our finding that HCWs from Asian and Black ethnic groups were more likely to report diabetes than those from White ethnic groups was expected, given the wealth of evidence from general population studies [28]. Evidence on the effects of migration status is more limited, but previous studies have suggested a lower risk of type 2 diabetes in second-generation compared to first-generation migrants in the UK general population [29]. Likewise, the prevalence of hypertension has been shown to be higher in South Asian and Black ethnic groups than White groups in the UK population, but our finding relating to a higher prevalence in migrants from these HCW ethnic groups compared to their UK-born counterparts is novel. The differences in asthma prevalence between ethnic and migrant groups have also been reported in other UK studies [30, 31]. For instance, a study between documented and undocumented migrants showed high asthma co-occurrence with diabetes, cardiovascular disease and Chronic Obstructive Pulmonary Disease (COPD) among documented migrants compared to undocumented migrants [32]. High asthma prevalence has also been reported in South Asian populations, especially males [30]. One study showed that although high asthma prevalence was observed in Black Caribbean, Mixed Black Caribbean, and White boys, shorter residence in the UK (≤5 years) was a protective factor for Black Caribbean and Black African boys [31]. This emphasises the higher likelihood of asthma in UK residents compared to recent overseas-born migrant groups.

As shown in Fig. S 3, certain combinations of co-occurring LTCs were more prevalent in the UK-born (e.g. anxiety/depression and hypertension/stroke), while other combinations were more common among migrants (e.g. diabetes/liver disease and hypertension/kidney disease). However, it is difficult to draw conclusions on patterns of co-occurrence by migration status given the limited numbers reporting each long-term condition in our sample. A large, England NHS population-based study by Kuan et al. based on electronic health records of nearly four million patients detected some differences in co-occurrence of health conditions by ethnicity but not migration status, such as an association between breast cancer and atrial fibrillation for Black individuals, [33] but as with our study, the causes of such correlations require further investigation.

We found MLTC prevalence to be 15% overall, which aligns with an estimated 16.4% from a smaller study conducted in the UK working-age population [34]. Kuan et al. reported higher multimorbidity prevalence estimates because researchers used a broader definition of co-occurrence of health conditions (for example, the inclusion of upper respiratory tract infections and definition of comorbidity as the accumulation of additional conditions to an index condition over an individual’s entire lifetime) [33]. We also present evidence that the prevalence of MLTCs differs according to ethnicity and migration status, with groups born overseas having lower odds of reporting MLTCs than their UK-born counterparts and White individuals having a higher prevalence than other ethnic groups (in agreement with Kuan et al.: prevalence of co-occurrence of two or more conditions was 78.7% for White, 60.1% for Black and 60.2% for South Asian individuals). Another study showed that migrants residing in the UK for more than 10 years had increased odds of indicating the presence of a long-standing illness compared to non-migrants; in contrast, these odds were lower for migrants in the UK for 10 years or less compared to non-migrants [35]. It is interesting to note that the differences we found by ethnicity and migration status became more pronounced after additional adjustment for health and lifestyle factors; this might be related to the mediating effect of these factors on the development of diabetes and hypertension (conditions that were generally found to be more prevalent in ethnic minority groups). Our results suggest that studies investigating disease and MLTC prevalence by ethnicity should take migrant status into account, as well as the length of stay in the country of residence. Our results suggest that, around the time of migration, migrant HCWs appear to have a lower risk of MLTCs compared to the HCWs born in the UK and that odds of reporting MLTCs increase with time since migration to become proportionate with UK-born HCWs. The same pattern is seen in general population studies investigating mortality in migrants in Western Europe [36, 37]. The mechanisms that underlie this observation have been hypothesised to include acculturation to the negative health behaviours of the host society and the disproportionate exposure of migrants, when compared to the native population, to adverse conditions (e.g. residence in more deprived areas) that might affect health [37].

Our study has some limitations. The main limitation is the absence of a control group, so we do not know if patterns of MLTCs differ between HCWs and non-HCW populations. Also, the list of health conditions included in the UK-REACH questionnaire was standardised using the Wellcome Trust COVID-19 questionnaire list of pre-existing health conditions [20]. The tool included three mental health conditions: depression, anxiety and psychiatric disorder; however, “psychiatric disorder” was poorly defined and was not included in our analysis. Some of the ethnicity/migration status subgroups are small (particularly “Other UK-born”), which has implications for the power to detect differences between these groups and the reference group. There may be reporting bias, considering the data presented are based on a cohort recruited for the UK-REACH study and self-reported, and migrants who have severe co-occurring LTCs working in the NHS may have chosen not to report to a questionnaire due to stigma. As with any observational questionnaire study, our results may be affected by selection and non-response bias. However, our sample is similar to the NHS in composition in terms of HCWs from ethnic minority groups (June 2022 UK Government estimate 13.1% Asian, 7.4% Black, 2.1% Mixed, 74.3% White and 3.0% Other [38]), HCWs born overseas (ONS 2019 data estimated 25% of hospital workers were born overseas [39] vs 26.8% in our sample) and in sex distribution (NHS workforce estimated 77% female in 2019 [40] vs 76% in our sample). Furthermore, our estimate of the prevalence of MLTCs (15.0%) aligns with a small study conducted in working-age populations in the UK (16.4%) [34]. Finally, since this is a cross-sectional analysis, we cannot be definitive about the direction of any reported associations. Nevertheless, a significant strength of our study comes from the richness of our data, which allows us to determine the contribution of some of these interrelated factors to the higher risk of MLTCs for HCWs from certain ethnic/migrant groups, with an emphasis on migration duration.

## Conclusions

Our findings suggest that the prevalence of common LTCs in UK HCWs is associated with ethnicity and migration status. Those from the White UK-born population may be at higher risk of mental health problems than those from other ethnic and migrant groups. Cardiometabolic conditions are reported more frequently in Asian and Black ethnic groups, with odds being affected by migration status. Migrants appeared to have lower odds of MLTCs than the White UK-born population, but these odds increased with an extended stay in the UK. Further research on LTCs/MLTCs in this population should include longitudinal studies with linkage to healthcare records and should collect information on both ethnicity and migration status. Interventions should be co-developed with HCWs from different ethnic and migrant groups, focussed upon patterns of conditions prevalent in specific HCW subgroups to reduce the overall burden of LTCs/MLTCs. In addition, optional organisational periodic health assessments could be considered to monitor the health of HCWs (these should include an assessment of BMI considering the prevalence of obesity in our cohort and other UK HCW cohorts [41]). Results of assessments could be fed back to each HCW, and bespoke support, reflective of their unique identity, could be provided to help modify individual health risks. Such personalised interventions would benefit not only individual HCWs but also healthcare organisations and the UK patient population as a whole.

### Supplementary Information

**Additional file 1:** **Text S1.** The UK-REACH study collaborative group. **Text S2.** List of participating healthcare regulators.

**Additional file 2:** **Table S1.** Derivation of covariates from UK-REACH baseline questionnaire data.

**Additional file 3:** **Figure S1.** Healthcare worker cohort recruitment flowchart.

**Additional file 4:** **Table S2.** Frequency and proportion of the five most frequently-reported long-term conditions, by ethnicity and migration status (*n*=12,100).

**Additional file 5:** **Figure S2.** A-E - The association of ethnicity and migration status with the five most prevalent long-term health conditions reported by healthcare workers in the UK-REACH study (as shown in Figure 1 main text, but here accompanied by text showing the adjusted odds ratio and 95% confidence interval).

**Additional file 6: Figure S3.** Heatmaps highlighting the pattern of prevalence of two long-term conditions.

**Additional file 7:** **Table S3.** The association of ethnicity, migration status, sociodemographic and health factors with long-term condition count (negative binomial regression).

## Data Availability

To access data or samples produced by the UK-REACH study, the working group representative must first submit a request to the Core Management Group by contacting the UK-REACH Project Manager in the first instance. For ancillary studies outside of the core deliverables, the Steering Committee will make final decisions once they have been approved by the Core Management Group. Decisions on granting the access to data/materials will be made within eight weeks. Third party requests from outside the Project will require explicit approval of the Steering Committee once approved by the Core Management Group. Note that should there be significant numbers of requests to access data and/or samples then a separate Data Access Committee will be convened to appraise requests in the first instance.

## Abbreviations

95%CI: 95% confidence interval
AHP: Allied Health Professional
aOR: Adjusted odds ratio
aRR: Adjusted rate ratio
BMI: Body mass index
CHERRIES: The Checklist for Reporting Results of Internet E-Surveys
COPD: Chronic obstructive pulmonary disease
HCW: Healthcare worker
IMD: Index of multiple deprivation
IQR: Interquartile range
LTCs: Long-term conditions
MLTCs: Multiple long-term conditions
NHS: National Health Service
ONS: The Office for National Statistics
OR: Odds ratio
PAI: Physical activity index
PTSD: Post-traumatic stress disorder
UK: United Kingdom
UK-REACH: The United Kingdom Research study into Ethnicity And COVID-19 diagnosis and outcomes in Healthcare workers
