# Supplementary material for: Association between ethnicity and migration status with the prevalence of single and multiple long-term conditions in UK healthcare workers
Source: BMC Med. 2023 Nov 30;21:433. doi: 10.1186/s12916-023-03109-w (PMC10688453; doi:10.1186/s12916-023-03109-w)
Supplement: Supplementary file 1 — Additional file 1: Text S1. The UK-REACH study collaborative group. Text S2. List of participating healthcare regulators. [file 12916_2023_3109_MOESM1_ESM.docx]

**Text S1. The UK-REACH study collaborative group**

Manish Pareek (Chief investigator, University of Leicester), Laura Gray (University of Leicester), Laura Nellums (University of Nottingham), Anna L Guyatt (University of Leicester), Catherine John (University of Leicester), I Chris McManus (University College London), Katherine Woolf (University College London), Ibrahim Abubakar (University College London), Amit Gupta (Oxford University Hospitals), Avinash Aujayeb (Northumbria Specialist Emergency Care Hospital), Bindu Gregary (Royal Preston Hospital), Rubina Reza (Derbyshire Healthcare NHS Foundation Trust), Sandra Simpson (Nottinghamshire Healthcare NHS Foundation Trust), Stephen Zingwe (Berkshire Healthcare NHS Foundation Trust), Keith R Abrams (University of York), Martin D Tobin (University of Leicester), Louise Wain (University of Leicester), Sue Carr (University Hospitals of Leicester NHS Trust), Edward Dove (University of Edinburgh), Kamlesh Khunti (University of Leicester), David Ford (University of Swansea), Robert Free (University of Leicester).

**Text S2. List of participating healthcare regulators**

General Medical Council, Nursing and Midwifery Council, General Dental Council, Health and Care Professions Council, General Optical Council, General Pharmaceutical Council, or the Pharmaceutical Society of Northern Ireland.
