# Supplementary material for: Association between ethnicity and migration status with the prevalence of single and multiple long-term conditions in UK healthcare workers
Source: BMC Med. 2023 Nov 30;21:433. doi: 10.1186/s12916-023-03109-w (PMC10688453; doi:10.1186/s12916-023-03109-w)
Supplement: Supplementary file 2 — Additional file 2: Table S1. Derivation of covariates from UK-REACH baseline questionnaire data. [file 12916_2023_3109_MOESM2_ESM.docx]

## Table S1. Derivation of covariates from UK-REACH baseline questionnaire data.

| **Variable** | **Description** |
| --- | --- |
| **Age** | Continuous variable. Age in years. Derived from date of birth entered by participants at registration. |
| **Sex** | Binary variable. Participants were asked their sex assigned at birth. |
| **Ethnicity** | Categorical variable. Participants were asked to select their ethnicity from a list of the 18 Office for National Statistics (O­­­NS) categories:  Asian/Asian British – Indian  Asian/Asian British – Pakistani  Asian/Asian British – Bangladeshi  Asian/Asian British – Chinese  Asian/Asian British - Any other Asian background  Black/African/Caribbean/Black British - African  Black/African/Caribbean/Black British – Caribbean  Black/African/Caribbean/Black British - Any other Black/African/Caribbean background Mixed/Multiple ethnic groups - White and Black Caribbean  Mixed/Multiple ethnic groups - White and Black African  Mixed/Multiple ethnic groups - White and Asian  Mixed/Multiple ethnic groups - Any other Mixed/multiple ethnic background  White - English/Welsh/Scottish/Northern Irish/British  White – Irish  White - Gypsy or Irish Traveller  White - Any other white background  Other ethnic group – Arab  Other ethnic group - Any other ethnic background  These were categorised into the 5 aggregated Office for National Statistics ethnicity categories (Asian, Black, Mixed, White, Other). |
| **Migration status** | Binary variable. Participants were asked whether they were born in the UK. |
| **Migration duration** | Ordinal variable. Participants who indicated they were not born in the UK were asked *“In which year did you move to the UK? If you are unsure, please give your best estimate.”*  Using this information, we calculated the number of years since a participant moved to the UK and derived a six level variable (‘born in the UK’, ‘0 to 5 years’, ‘5 to 10 years’, ‘10 to 15 years’, ‘15 to 20 years’ and ‘> 20 years’) |
| **Ethnicity and migration status combined** | Categorical variable. 18 ONS ethnicity categories reduced to five aggregated ethnicity categories (as also used by the ONS): White, Asian, Black, Mixed, Other, stratified by migration status (UK-born and overseas-born). |
| **Index of Multiple Deprivation (IMD) quintile** | Ordinal variable. Participants provided their residential postcode on registration for the study. This was used to determine the Index of Multiple Deprivation (the official measure of deprivation for small areas of England) in the area in which they live. The IMD ranks all areas in England based on 7 measures of deprivation and the ranks can be expressed as quintiles. Lower quintiles indicate more deprivation. Although Wales, Scotland and Northern Ireland have their own measures of deprivation, these are said not to be directly comparable to English IMD and therefore we elected to impute an ‘English IMD’ for residents of the these nations.  **Source:** [https://www.gov.uk/government/statistics/english-indices-of-deprivation-2019](about:blank) |
| **Smoking status** | Binary variable. Participants were asked to indicate their current smoking status.  Three level categorical variable (never smoker, ex-smoker and current smoker).Never and ex-smokers were grouped together and compared with current smokers. |
| **Body Mass Index (BMI)** | Ordinal variable. Participants were asked to input their height and weight. These were used to calculate BMI in kg/m^2^. We then derived a 6 level ordinal variable using ethnicity-specific thresholds recommended by NICE: underweight, healthy weight, overweight, obesity class 1, obesity class 2 and obesity class 3.  **Source**: [https://www.nice.org.uk/guidance/cg189/chapter/Recommendations#identifying-and-assessing-overweight-obesity-and-central-adiposity](about:blank#identifying-and-assessing-overweight-obesity-and-central-adiposity) |
| **Alcohol** | Ordinal variable. Participants were asked about their weekly consumption of alcohol in units selecting from the following categories: ‘none’, ‘1 to 7’, ‘8 to 14’, ‘15 to 21’, ‘22 to 28’, ’29 to 35’, 36 to 50’ and ‘≥51’.  Due to low numbers of participants reporting consumption of >21 units per week we collapsed these categories into one resulting in a five-level variable. |
| **Physical activity index (PAI)** | Ordinal variable. 4 levels (inactive, moderately inactive, moderately active, active). Derived from the completion of the General Practice Physical Activity Questionnaire (GPPAQ) – a validated screening tool used in primary care to assess the physical activity levels of adults  **Source**: [https://www.nice.org.uk/guidance/cg61/evidence/appendix-j-gppaq-pdf-196701669](about:blank) |
| **Occupation** | Categorical variable. Participants were asked to select their main job/role. Categorised as below:  **Doctor or medical support** – Doctor, Advanced Critical Care Practitioner, Anaesthesia associate, Surgical Care Practitioner, Other medical associate  **Nurse, NA or Midwife –** Advanced Nurse Practitioner, Healthcare assistant, Maternity support worker, Midwife, Nurse, Nursing Associate, Other nursing and midwifery role,  **Allied Health Professional (including pharmacists, healthcare scientists, ambulance workers and those in optical roles)** - Arts therapist, Biomedical scientist, Chiropodist/Podiatrist, Clinical scientist, Dietician, Hearing aid dispenser, Occupational therapist, Operating department practitioner, Orthoptist, Physiotherapist, Practitioner psychologist, Prosthetist / Orthotist, Radiographer, Speech and language therapist, Other Allied Health Professional role, Emergency medical , Paramedic , Other ambulance role, OT Support , Phlebotomist, Physiotherapy Assistant, Radiography Other clinical support role , Pharmacist , Pharmacy technician, Other pharmacy role, Optical - Dispensing optician, Optometrist, Other Optical role  **Dental –** Clinical dental technician, Dental Hygienist, Dental nurse, Dental technician, Dentist, Other dental role  **Admin, estates or other –** Administration, Catering services, Domestic services, Estates services, Porter, Other |
| **Long-term conditions** (Pre-existing health conditions) | Binary variables. Participants were asked to indicate if they had the following pre-existing health conditions:  1, Organ transplant  2, Diabetes (Type I or II)  3, Heart disease or heart problems  4, Hypertension  5, Overweight  6, Stroke  7, Kidney disease  8, Liver disease  9, Anaemia  10, Asthma  11, Other lung condition such as COPD, bronchitis or emphysema  12, Cancer  13, Condition affecting the brain and nerves (e.g., Dementia, Parkinson's, Multiple Sclerosis)  14, A weakened immune system or reduced ability to deal with infections (as a result of a disease or treatment)  15, Depression  16, Anxiety  17, Psychiatric disorder |
| **Long-term conditions (count)** | Continuous variable (count). We used information from the long term conditions variable above to derive a variable that represented a count of the number of long-term conditions a participant reported. We did not include ‘overweight’, ‘anaemia’ or ‘psychiatric disorder’ in the count for reasons explained in the methods. |
| **Multiple long-term conditions (binary)** | Binary variable. We used the ‘long-term conditions count’ variable described above to derive a binary variable indicating whether a participant reported ≥2 long-term conditions or < 2 long-term conditions. |
