# Supplementary material for: Association between ethnicity and migration status with the prevalence of single and multiple long-term conditions in UK healthcare workers
Source: BMC Med. 2023 Nov 30;21:433. doi: 10.1186/s12916-023-03109-w (PMC10688453; doi:10.1186/s12916-023-03109-w)
Supplement: Supplementary file 3 — Additional file 3: Figure S1. Healthcare worker cohort recruitment flowchart. [file 12916_2023_3109_MOESM3_ESM.docx]

## Figure S1. Healthcare worker cohort recruitment flowchart.

* There were 15,997 views of the questionnaire, 155 duplicate records were removed, leaving 15,842 unique HCW views. † Corresponds to an effective response rate of 57.1% of those who registered/created a profile on the study website (and 84.5% of those who consented, 1.4% of those who were sent an email, and 3.2% of those who opened the email). HCW – healthcare worker.
