# Supplementary material for: Association between ethnicity and migration status with the prevalence of single and multiple long-term conditions in UK healthcare workers
Source: BMC Med. 2023 Nov 30;21:433. doi: 10.1186/s12916-023-03109-w (PMC10688453; doi:10.1186/s12916-023-03109-w)
Supplement: Supplementary file 4 — Additional file 4: Table S2. Frequency and proportion of the five most frequently-reported long-term conditions, by ethnicity and migration status (n=12,100). [file 12916_2023_3109_MOESM4_ESM.docx]

## Table S2. Frequency and proportion of the five most frequently-reported long-term conditions, by ethnicity and migration status (n=12,100).

|  |  | **Number reporting one of the five most commonly-reported long-term conditions (%)** | | | | |
| --- | --- | --- | --- | --- | --- | --- |
| **Ethnicity and migration status** | **Total** | **Anxiety** | **Depression** | **Asthma** | **Diabetes** | **Hypertension** |
| White UK-born | 7,444 | 1,310 (17.6) | 979 (13.2) | 967 (13.0) | 243 (3.3) | 610 (8.2) |
| White overseas-born | 1,048 | 157 (15.0) | 96 (9.2) | 93 (8.9) | 30 (2.9) | 73 (7.0) |
| Asian UK-born | 834 | 100 (12.0) | 69 (8.3) | 121 (14.5) | 26 (3.1) | 34 (4.1) |
| Asian overseas-born | 1,492 | 102 (6.8) | 61 (4.1) | 119 (8.0) | 132 (8.8) | 190 (12.7) |
| Black UK-born | 152 | 16 (10.5) | 10 (6.6) | 24 (15.8) | 10 (6.6) | 18 (11.8) |
| Black overseas-born | 369 | 23 (6.2) | 13 (3.5) | 28 (7.6) | 18 (4.9) | 64 (17.3) |
| Mixed UK-born | 383 | 57 (14.9) | 41 (10.7) | 74 (19.3) | 11 (2.9) | 24 (6.3) |
| Mixed overseas-born | 130 | 16 (12.3) | 13 (10.0) | 14 (10.8) | 7 (5.4) | 16 (12.3) |
| Other UK-born | 45 | 6 (13.3) | 4 (8.9) | 10 (22.2) | 1 (2.2) | 5 (11.1) |
| Other overseas-born | 203 | 17 (8.4) | 10 (4.9) | 21 (10.3) | 8 (3.9) | 22 (10.8) |

Freq – frequency
