# Supplementary material for: Association between ethnicity and migration status with the prevalence of single and multiple long-term conditions in UK healthcare workers
Source: BMC Med. 2023 Nov 30;21:433. doi: 10.1186/s12916-023-03109-w (PMC10688453; doi:10.1186/s12916-023-03109-w)
Supplement: Supplementary file 5 — Additional file 5: Figure S2. A-E - The association of ethnicity and migration status with the five most prevalent long-term health conditions reported by healthcare workers in the UK-REACH study (as shown in Figure 1 main text, but here accompanied by text showing the adjusted odds ratio and 95% confidence interval). [file 12916_2023_3109_MOESM5_ESM.docx]

## **Figure S2.** The association of ethnicity and migration status with the five most prevalent long-term health conditions reported by healthcare workers in the UK-REACH study (as shown in Figure 1 main text, but here accompanied by text showing the adjusted odds ratio and 95% confidence interval): Panel A) anxiety, B) depression, C) hypertension, D) diabetes and E) asthma.

## Associations are derived from logistic regression models presented as odds ratios (circles) and 95% confidence intervals (spikes bars). White UK-born is the reference group. Odds ratios are adjusted for age, sex, index of multiple deprivation quintile and occupation. N=12,100 for all models. Panel D results for the association between the “Other UK-born” group and diabetes is omitted due to low numbers (<5) in this group producing a very wide 95% confidence interval range (0.07–4.13). Ref – reference group.

A) Anxiety

B) Depression

C) Hypertension

D) Diabetes

E) Asthma
