# Supplementary material for: Association between ethnicity and migration status with the prevalence of single and multiple long-term conditions in UK healthcare workers
Source: BMC Med. 2023 Nov 30;21:433. doi: 10.1186/s12916-023-03109-w (PMC10688453; doi:10.1186/s12916-023-03109-w)
Supplement: Supplementary file 6 — Additional file 6: Figure S3. Heatmaps highlighting the pattern of prevalence of two long-term conditions. [file 12916_2023_3109_MOESM6_ESM.docx]

## **Figure S3. Heatmaps highlighting the pattern of prevalence of two long-term conditions** for a) UK-born healthcare worker respondents and b) overseas-born healthcare worker respondents. Numbers represent the percentage of respondents reporting the condition on the y-axis that also reported the condition on the x-axis.
