# Supplementary material for: Association between ethnicity and migration status with the prevalence of single and multiple long-term conditions in UK healthcare workers
Source: BMC Med. 2023 Nov 30;21:433. doi: 10.1186/s12916-023-03109-w (PMC10688453; doi:10.1186/s12916-023-03109-w)
Supplement: Supplementary file 7 — Additional file 7: Table S3. The association of ethnicity, migration status, sociodemographic and health factors with long-term condition count (negative binomial regression). [file 12916_2023_3109_MOESM7_ESM.docx]

## Table S3. The association of ethnicity, migration status, sociodemographic and health factors with long-term condition count (negative binomial regression): A) adjusted for ethnicity, migration status and sociodemographic covariates only, and B) additionally adjusted for health and lifestyle covariates (n=12,100).

| **Variable** | **A)** |  |  | **B)** |  |
| --- | --- | --- | --- | --- | --- |
|  | **Adjusted rate ratio (95% CI)** | **P value** |  | **Adjusted rate ratio (95% CI)** | **P value** |
| **Ethnicity/migration status**  White UK  White overseas  Asian UK  Asian overseas  Black UK  Black overseas  Mixed UK  Mixed overseas  Other UK  Other overseas | Ref  0.86 (0.78 – 0.95)  0.91 (0.81 – 1.03)  0.86 (0.78 – 0.94)  0.96 (0.76 – 1.21)  0.74 (0.62 – 0.87)  1.13 (0.97 – 1.30)  1.02 (0.79 – 1.31)  1.20 (0.80 – 1.80)  0.80 (0.64 – 1.00) | -  0.003  0.13  0.001  0.71  <0.001  0.12  0.90  0.38  0.06 |  | Ref  0.88 (0.80 – 0.97)  0.85 (0.75 – 0.95)  0.79 (0.72 – 0.87)  0.81 (0.65 – 1.02)  0.63 (0.53 – 0.75)  1.10 (0.95 – 1.27)  0.96 (0.75 – 1.23)  1.06 (0.72 – 1.58)  0.71 (0.57 – 0.89) | -  0.009  0.004  <0.001  0.08  <0.001  0.20  0.73  0.76  0.003 |
| **Age**, per decade increase | 1.12 (1.09 – 1.14) | <0.001 |  | 1.10 (1.07 – 1.12) | <0.001 |
| **Sex**  Male  Female | Ref  0.88 (0.82 – 0.94) | -  <0.001 |  | Ref  0.90 (0.84 – 0.96) | -  0.001 |
| **Occupation**  Medical  Nursing  Allied Health Professional*  Dental  Admin/estates/other | Ref  1.69 (1.54 – 1.85)  1.18 (1.09 – 1.28)  1.18 (1.04 – 1.34)  1.47 (1.30 – 1.67) | -  <0.001  <0.001  0.01  <0.001 |  | Ref  1.41 (1.28 – 1.54)  1.12 (1.03 – 1.21)  1.09 (0.96 – 1.24)  1.21 (1.06 – 1.37) | -  <0.001  0.008  0.19  0.004 |
| **IMD quintile**  1 (most deprived)  2  3  4  5 (least deprived) | 1.07 (0.96 – 1.19)  0.99 (0.90 – 1.08)  Ref  0.90 (0.83 – 0.98)  0.84 (0.78 – 0.91) | 0.20  0.83  -  0.01  <0.001 |  | 1.01 (0.92 – 1.12)  0.97 (0.89 – 1.06)  Ref  0.93 (0.86 – 1.00)  0.88 (0.82 – 0.95) | 0.81  0.51  -  0.05  0.002 |
| **Body mass index (kg/m^2^)†**  Underweight  Healthy weight  Overweight  Obesity class 1  Obesity class 2  Obesity class 3 |  |  |  | 1.03 (0.79 – 1.34)  Ref  1.31 (1.22 – 1.40)  1.65 (1.52 – 1.78)  1.95 (1.75 – 2.17)  2.16 (1.89 – 2.47) | 0.84  -  <0.001  <0.001  <0.001  <0.001 |
| **Physical activity index**  Active  Moderately active  Moderately inactive  Inactive |  |  |  | Ref  1.13 (1.05 – 1.22)  1.17 (1.09 – 1.26)  1.32 (1.23 – 1.42) | -  <0.001  <0.001  <0.001 |
| **Smoking status**  Never smoker  Ex-smoker  Current smoker |  |  |  | Ref  1.13 (1.06 – 1.20)  1.37 (1.23 – 1.52) | -  <0.001  <0.001 |
| **Units of alcohol per week**  None  1 – 7  8 – 14  15 – 21  >28 |  |  |  | Ref  0.84 (0.79 – 0.90)  0.84 (0.77 – 0.91)  0.81 (0.73 – 0.90)  0.95 (0.84 – 1.07) | -  <0.001  <0.001  <0.001  0.39 |

* includes those working in pharmacy, optical, healthcare scientist and ambulance roles; †using ethnicity-specific cut-offs (see Methods for details).

95% CI – 95% confidence interval; IMD – index of multiple deprivation; Ref – reference group.
